# SGTA interacts with the proteasomal ubiquitin receptor Rpn13 via a carboxylate clamp mechanism

**DOI:** 10.1038/srep36622

**Published:** 2016-11-09

**Authors:** Arjun Thapaliya, Yvonne Nyathi, Santiago Martínez-Lumbreras, Ewelina M. Krysztofinska, Nicola J. Evans, Isabelle L. Terry, Stephen High, Rivka L. Isaacson

**Affiliations:** 1 Department of Chemistry, King’s College London, Britannia House, Trinity Street, London, SE1 1DB, U.K; 2 School of Biological Sciences, Faculty of Biology, Medicine and Health, University of Manchester, The Michael Smith Building, Oxford Road, Manchester, M13 9PT, U.K

## Abstract

The fate of secretory and membrane proteins that mislocalize to the cytosol is decided by a collaboration between cochaperone SGTA (small, glutamine-rich, tetratricopeptide repeat protein alpha) and the BAG6 complex, whose operation relies on multiple transient and subtly discriminated interactions with diverse binding partners. These include chaperones, membrane-targeting proteins and ubiquitination enzymes. Recently a direct interaction was discovered between SGTA and the proteasome, mediated by the intrinsic proteasomal ubiquitin receptor Rpn13. Here, we structurally and biophysically characterize this binding and identify a region of the Rpn13 C-terminal domain that is necessary and sufficient to facilitate it. We show that the contact occurs through a carboxylate clamp-mediated molecular recognition event with the TPR domain of SGTA, and provide evidence that the interaction can mediate the association of Rpn13 and SGTA in a cellular context.

The cytosol of eukaryotes is crowded and, to function successfully, our cells employ quality control mechanisms that deal with a range of misfolded and mislocalized proteins^1^,^2^. In the case of membrane and secretory proteins that mislocalize to the cytosol, collectively termed MLPs, their fate is determined by a specialised quality control pathway that employs SGTA (small, glutamine-rich, tetratricopeptide repeat protein alpha) and the heterotrimeric BAG6 (BCL2-associated athanogene 6) complex^2^,^3^. SGTA and the BAG6 complex were initially discovered through their role in the biogenesis of tail-anchored membrane proteins and subsequently shown to regulate the ubiquitination and proteasomal degradation of MLPs^2^,^3^. The role of SGTA and BAG6 in both processes relies on multiple transient, and subtly discriminated, interactions with diverse binding partners and effectors^4^,^5^,^6^,^7^,^8^,^9^,^10^. These interactions contribute to a proposed BAG6/SGTA quality control cycle that can direct hydrophobic substrates towards either ubiquitination and proteasomal degradation (MLPs) or membrane insertion (tail-anchored proteins)^2^,^3^,^4^,^5^,^11^,^12^,^13^. The BAG6 complex comprises at least two copies each of BAG6, UBL4A (ubiquitin-like protein 4A) and TRC35 (transmembrane recognition complex 35) and displays two different kinds of UBL domains; UBL4A_UBL and BAG6_UBL^11^,^14^,^15^. SGTA, on the other hand, is a homodimer, tightly constrained at its N-terminal domain^7^,^16^,^17^. It has a central TPR domain capable of direct interaction with both Hsp70 and Hsp90 chaperones, proteasomal subunits and a variety of disease-related proteins^6^,^18^, and a C-terminal domain capable of binding hydrophobic substrates^19^. Within this SGTA/BAG6 quality control cycle, BAG6 identifies hydrophobic MLPs and recruits an E3 ubiquitin ligase, RNF126, thereby facilitating their selective ubiquitination and entry into the pathway for proteasomal degradation^8^,^13^. Conversely, SGTA promotes substrate deubiquitination, thereby delaying the proteasomal degradation of MLPs^4^,^5^.

The 26S proteasome is the cellular machinery responsible for a substantial proportion of protein degradation^20^. It consists of a barrel shaped 20S proteolytic core particle, with its ends capped by 19S regulatory particles^20^. Rpn10 and Rpn13 are two intrinsic proteasomal ubiquitin receptors, present at the 19S regulatory particle, that selectively recognise ubiquitinated substrates^21^,^22^. Furthermore, whilst the N-terminal region of BAG6 associates with Rpn10^12^,^23^, the central TPR domain of SGTA was recently shown to bind to the C-terminal domain of Rpn13^24^. These observations led to the proposal that the BAG6/SGTA quality control cycle may be operational at the 19S regulatory particle of the proteasome, thereby regulating the access of MLPs to the proteolytic core^24^. Rpn13 consists of an N-terminal pleckstrin-like receptor for ubiquitin (PRU) domain that binds to both ubiquitin and the proteasome, a conserved C-terminal DEUBiquitinase ADaptor (DEUBAD) domain that binds to the deubiquitinating enzyme UCH37 (UCH-L5) and, lastly, a flexible C-terminal extension^25^. Rpn13 has reduced affinity for ubiquitin in its proteasome-unbound state as a consequence of an interaction between its PRU and DEUBAD domains^25^. Binding to the proteasome abrogates this interaction and results in Rpn13 activation^25^. Upon activation, the UCH37 deubiquitinase can bind to the Rpn13 DEUBAD domain in a manner that allows UCH37 access to its ubiquitinated substrates^26^,^27^. Therefore in the context of the BAG6/SGTA cycle, it has been speculated that the interaction between SGTA and Rpn13 provides SGTA-bound substrates with access to the UCH37 deubiquitinase, and could thereby facilitate a rescue option for prematurely ubiquitinated substrates^24^,^25^,^26^,^27^.

TPR domains consist of a number of helix-turn-helix repeat motifs, each made of 34 amino acid residues that fold into two anti-parallel alpha helices, and are well known as mediators of protein-protein interactions^28^,^29^. These repeat motifs are arranged in tandem to form a concave surface, referred to as the TPR groove, which acts as a binding site for polypeptides from interacting proteins. In the case of co-chaperones such as STIP1 (or HOP), the binding of the TPR groove to the conserved C-terminal MEEVD/IEEVD motif of Hsp70s and Hsp90s is dependent upon conserved basic residues that line the TPR groove and mediate electrostatic interactions with this motif 30,31. It is these interactions that give rise to the two-carboxylate clamp mode of molecular recognition, in which free C-terminal carboxylates, present on the main chain and side chain of the final aspartate residue of these Hsps, form tight interactions with basic residues present in the TPR groove^31^,^32^. The TPR domain of SGTA has three similar repeat motifs arranged in tandem followed by a C-terminal capping helix that packs against the third TPR repeat. These seven helices assemble into a right-handed super-helical fold that accommodates the peptide-binding groove detailed above^6^,^18^.

In this study we first characterize the direct interaction between the central TPR domain of SGTA and the C-terminal domain of Rpn13 using solution NMR spectroscopy, size exclusion chromatography and isothermal titration calorimetry. We identify a region of the Rpn13 C-terminal domain that is necessary and sufficient to facilitate its interaction with SGTA *in vitro*. We use site-directed mutagenesis to confirm that binding occurs via a carboxylate clamp mechanism and employ paramagnetic spin labelling to model the resulting Rpn13 derived peptide complex with the TPR domain of SGTA. Lastly, we use a cell culture based model to show that the carboxylate clamp dependent binding of an Rpn13 C-terminal fragment is essential to out-compete endogenous proteasomal Rpn13 for binding to overexpressed SGTA and thereby offset its ability to increase the steady state level of a previously defined model MLP. Taken together these data provide molecular level characterisation of the SGTA-Rpn13 interaction which illuminates an emerging area of protein quality control in the cytoplasm.

## Results

### Biophysical characterization of the SGTA-Rpn13 interaction

**Figure 1:**
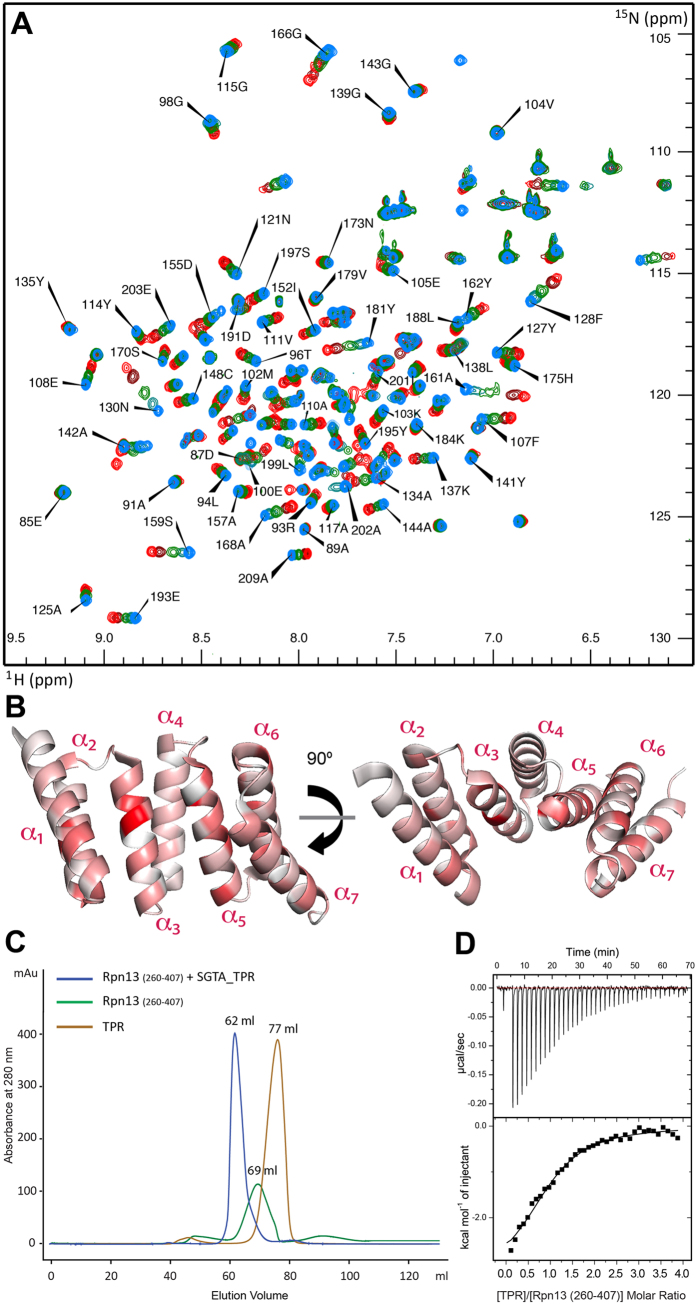
SGTA_TPR/Rpn13_260-407_ interaction studies. (**A**) ^1^H-^15^N HSQC spectra of ^15^N-labelled SGTA_TPR at different titration points with unlabelled Rpn13_260-407_ (1:0, 1:1, 1:2, 1:3, 1:8; in blue, teal, green, maroon, and red respectively). (**B**) Orthogonal cartoon views of SGTA_TPR (PDB accession code 2VYI)^18^ coloured according to normalised chemical shift perturbation (CSP) Δδ^av^ values upon binding to Rpn13_260-407_ with most perturbed residues shown in red. (**C**) Size exclusion chromatography (SEC) experiments showing the formation of a stable complex as evident by co-elution of SGTA_TPR with Rpn13_260-407_. (**D**) ITC data showing binding of SGTA_TPR to Rpn13_260-407_. The thermodynamic binding constant of this interaction as determined by ITC was, K_d_ = 16.1 ± 1.4 μM.

**Figure 2:**
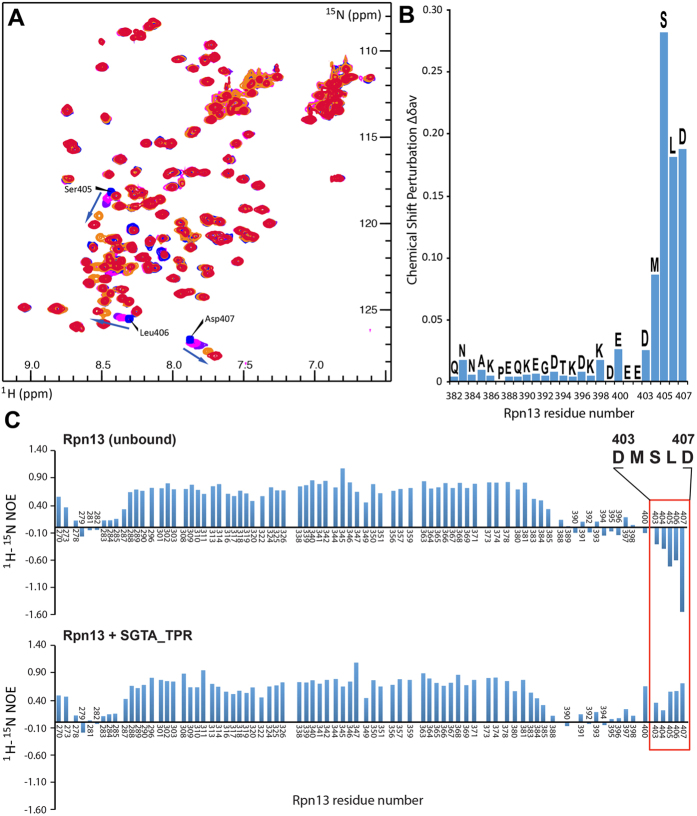
The extreme C-terminus of Rpn13_260-407_ is the SGTA_TPR interacting region. (**A**) ^1^H-^15^N HSQC spectra of ^15^N-labelled Rpn13_260-407_ at different titration points with unlabelled SGTA_TPR (1:0, 1:0.2, 1:0.5, 1:0.7, 1:1; in blue, light green, green, maroon, and red respectively). (**B**) Chemical shift perturbation (CSP) Δδ^av^ values derived from (**A**) of Rpn13 C-terminal residues 382–407 upon binding to SGTA_TPR. (**C**) ^1^H-^15^N heteronuclear NOE enhancement values of Rpn13 C-terminal residues in unbound and SGTA_TPR bound states.

In order to understand molecular details underlying the interaction between SGTA and Rpn13, solution NMR studies were carried out on excised domains of SGTA and Rpn13 guided by earlier mapping studies^24^. Reciprocal chemical shift perturbation (CSP) experiments were performed by titrating unlabelled Rpn13 C-terminal domain (residues 260–407; hereafter described as Rpn13_260-407_) into ^15^N-labelled TPR domain of SGTA (residues 84–211; hereafter referred to as SGTA_TPR) and vice versa. Assignments of backbone amide resonances were obtained from the BMRB (accession numbers 5709 and 17286, for SGTA_TPR and Rpn13_260-407_, respectively). The ^1^H-^15^N HSQC spectra of both SGTA_TPR and Rpn13_260-407_ show binding in a fast exchange regimen (Figs 1A and 2A). Widespread perturbation of backbone amide signals can be observed in ^1^H-^15^N HSQC spectra of ^15^N-labelled SGTA_TPR upon binding to Rpn13_260-407_ (Fig. 1A,B). However, from the Rpn13_260-407_ perspective, backbone amide peaks corresponding to only extreme C-terminal residues Met 404, Ser 405, Leu 406 and Asp 407 are perturbed upon binding to SGTA_TPR (Fig. 2A,B). Labelled Rpn13_260-407_ approaches saturation with a 1:1 addition of SGTA_TPR, with the CSP data yielding a K_d_ of 3.27 ± 0.87 μM (Figure S1). However, in the reciprocal titration SGTA_TPR amide peaks continue to shift even after eight equivalents of Rpn13_260-407_ have been added and the data cannot be fitted to a meaningful binding isotherm. Binding was further confirmed by size-exclusion chromatography (SEC) and isothermal titration calorimetry (ITC) (Fig. 1C,D). SGTA_TPR and Rpn13_260-407_ were mixed in a 1:1 molar ratio and analysed by SEC, with the resulting elution volume clearly indicative of a stable complex compared to the elution volumes of SGTA_TPR and Rpn13_260-407_ when run individually under identical conditions (Fig. 1C). ITC experiments yield a dissociation constant of 16.1 ± 1.4 μM in keeping with the value obtained from NMR. The favourable enthalpy and entropy values obtained from ITC (ΔH = −3.37 ± 0.08 kcal/mol; ΔS = 10.6 ± 0.8 cal/mol·K) suggest that binding between Rpn13_260-407_ and SGTA_TPR is driven by the formation of hydrogen bonds together with hydrophobic interactions (Fig. 1D).

### The extreme C-terminal region of Rpn13 is highly dynamic and transitions to a more ordered state upon binding to SGTA_TPR

To study the dynamic properties of free and SGTA_TPR-bound Rpn13_260-407_, ^1^H- ^15^N heteronuclear NOE experiments were carried out with a four second saturation transfer or control period. The extreme C-terminal region of free Rpn13 (residues 403–407) was found to be highly dynamic, with negative heteronuclear NOE enhancement values spanning this region of the amino acid sequence (Fig. 2C, upper panel). Upon addition of SGTA_TPR these values changed significantly indicating a loss of high frequency motions (Fig. 2C, lower panel). The average heteronuclear NOE enhancement value for Rpn13 residues Asp 403 to Asp 407 in the free and SGTA_TPR-bound state were −0.71 and 0.48, respectively, thus indicating the transition of the Rpn13 extreme C-terminal pentapeptide region to a more ordered state upon binding to SGTA_TPR (Fig. 2C). Taken together with the chemical shift perturbation study, these data clearly implicate the Rpn13 extreme C-terminal residues spanning Asp 403 to Asp 407 as the SGTA_TPR binding region of Rpn13_260-407_.

### The Rpn13 extreme C-terminal pentapeptide (DMSLD) is necessary and sufficient for its interaction with SGTA_TPR

**Figure 3:**
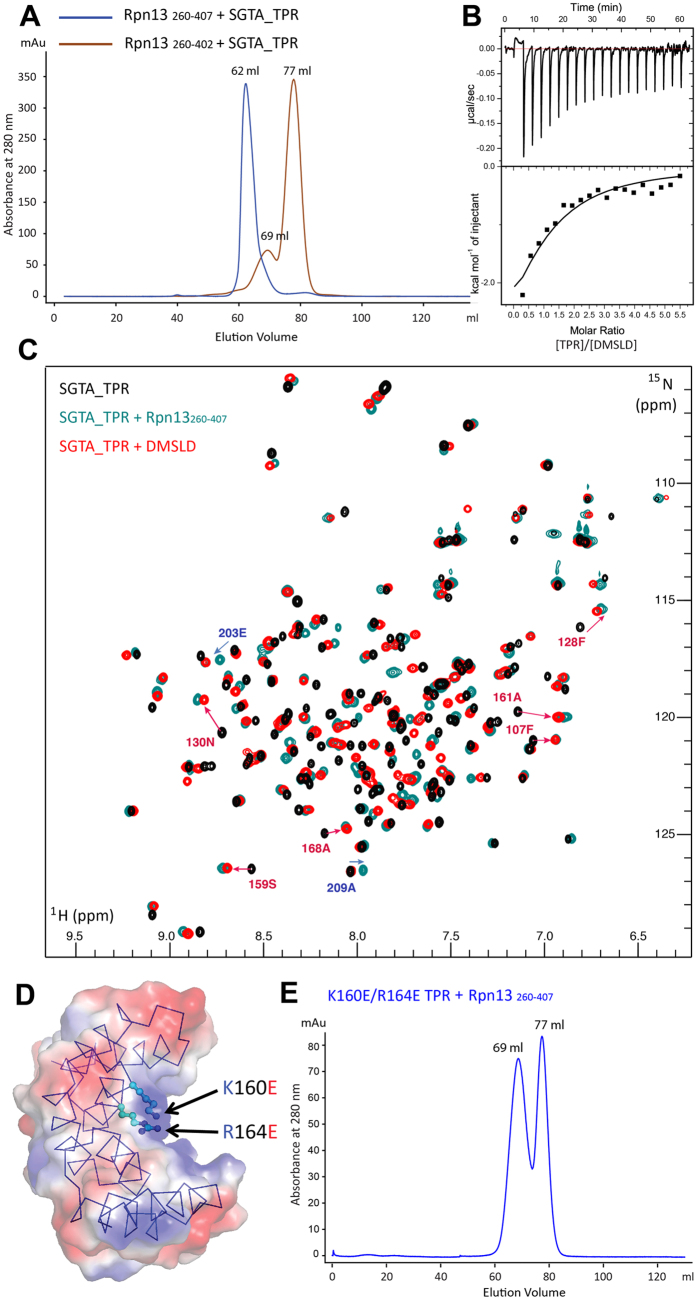
The Rpn13 C-terminal derived DMSLD pentapeptide is necessary and sufficient for its interaction with SGTA_TPR and is mediated via a carboxylate clamp of its terminal aspartate. (**A**) SEC experiments showing that lacking Rpn13 residues 403–407 is sufficient to abolish the SGTA_TPR/Rpn13_260-407_ interaction. (**B**) ITC data showing binding of SGTA_TPR to the Rpn13 extreme C-terminal derived DMSLD pentapeptide. The binding constant as determined by ITC for this interaction was, K_d_ = 71.9 ± 13.3 μM. (**C**) Overlay of ^1^H-^15^N HSQC spectra of ^15^N-labelled SGTA_TPR in its unbound form (black), and bound to a 3-fold molar excess of unlabelled Rpn13_260-407_ (green), or to the DMSLD pentapeptide (red). Backbone amide signals corresponding to residues with highest CSP Δδ^av^ values upon binding to either Rpn13_260-407_ or to the DMSLD pentapeptide are indicated with red arrows. Signals corresponding to amides only affected upon binding to Rpn13_260-407_ but not perturbed by the SGTA_TPR/DMSLD interaction are indicated with blue arrows. (**D**) SGTA_TPR crystal structure (PDB accession code 2VYI)^18^ with conserved basic residues Lys-160 and Arg-164 present at the TPR groove highlighted as sticks. (**E**) SEC experiments demonstrating that the K160E/R164E double mutant SGTA_TPR does not co-elute with Rpn13_260-407_ when mixed, even with Rpn13_260-407_ present at a 3-fold molar excess over SGTA_TPR.

Informed by CSP and ^1^H-^15^N heteronuclear NOE experiments, the role played by the extreme C-terminal pentapeptide (DMSLD; residues 403–407) of Rpn13 in facilitating the SGTA_TPR-Rpn13_260-407_ interaction was investigated by SEC, solution NMR spectroscopy and ITC. To this end, an Rpn13 construct devoid of this C-terminal pentapeptide (residues 260–402; hereafter referred to as Rpn13_260-402_) combined with SGTA_TPR in a 1:1 molar ratio and analysed by SEC showed no evidence of an interaction (Fig. 3A). Similarly, CSP experiments carried out by titrating unlabelled Rpn13_260-402_ into ^15^N-labelled SGTA_TPR with up to a 6-fold molar excess of Rpn13_260-402_ resulted in no perturbation of SGTA_TPR backbone amide signals (Figure S2). Additionally, ITC experiments carried out with Rpn13_260-402_ confirmed abrogation of binding with SGTA_TPR (Figure S3). Furthermore, the DMSLD peptide titrated into ^15^N-labelled SGTA_TPR exhibited a very similar pattern of CSPs amongst the most perturbed backbone amide signals lining the central TPR groove. In particular, SGTA_TPR residues Phe 107, Phe 128, Asn 130, Ser 159, Ala 161, and Ala 186 show similar CSPs when compared to ^1^H-^15^N HSQC spectra of SGTA_TPR upon binding to Rpn13_260-407_ at equivalent molar ratios (Figs 3C and S4). Interestingly, amide signals corresponding to residues 203–209 of helix-7 at the C-terminus of the SGTA_TPR construct were not affected by binding to the DMSLD peptide, however these signals are perturbed upon binding to Rpn13_260-407_ (Fig. 3C). Thus C-terminal residues of SGTA_TPR helix-7, particularly ones that do not structurally contribute to the groove, in turn do not form part of the peptide binding region. This strongly indicates the central TPR groove as the DMSLD peptide binding site. In addition, ITC experiments give a dissociation constant of 71.9 ± 13.3 μM with favourable enthalpy and entropy values (ΔH = −4.97 ± 0.41 kcal/mol; ΔS = 2.34 ± 0.04 cal/mol·K) suggestive of complex formation between SGTA_TPR and the DMSLD peptide driven by the formation of hydrogen bonds and hydrophobic interactions (Fig. 3B). These investigations confirm that the extreme C-terminal DMSLD pentapeptide is the region of Rpn13 that is necessary and sufficient for its interaction with SGTA_TPR.

### SGTA-Rpn13 interaction occurs via a two-carboxylate clamp mode of molecular recognition

The ‘two-carboxylate clamp’ mode of binding is a well-characterised mechanism of molecular recognition by TPR domains in the context of their binding to the conserved C-terminal IEEVD and MEEVD motifs of Hsp70 and Hsp90 chaperones, respectively^30^,^31^. It is known that conserved residues present on the concave surface of the TPR domain form a positively charged region within this groove that clamp, the two carboxylate groups of the terminal aspartate (Fig. 3D). As the Rpn13 amino acid sequence ends with an aspartate, and given the C-terminal DMSLD peptide being necessary and sufficient for this interaction, the possibility of a two-carboxylate clamp mechanism was explored by site-directed mutagenesis experiments. Structure based alignments suggest Lys 160 and Arg 164 as key residues forming such a clamp in SGTA_TPR^33^. Therefore a K160E/R164E double mutant version of SGTA was used in SEC and NMR experiments to understand the effect it had on Rpn13_260-407_ binding. SEC analysis in which the K160E/R164E double mutant SGTA_TPR was mixed with Rpn13_260-407_ showed no evidence of complex formation, even with Rpn13_260-407_ being present in a 3-fold molar excess (Fig. 3E). Reciprocal CSP experiments titrating unlabelled Rpn13_260-407_ into ^15^N-labelled K160E/R164E double mutant SGTA_TPR with up to a 6-fold molar excess of Rpn13_260-407_ and vice versa confirmed disruption of the SGTA_TPR-Rpn13_260-407_ interaction by the double mutant SGTA_TPR (Figures S5 and S6), indicative of a two-carboxylate clamp mechanism of binding.

### SGTA_TPR/Rpn13 derived DMSLD peptide complex

**Figure 4:**
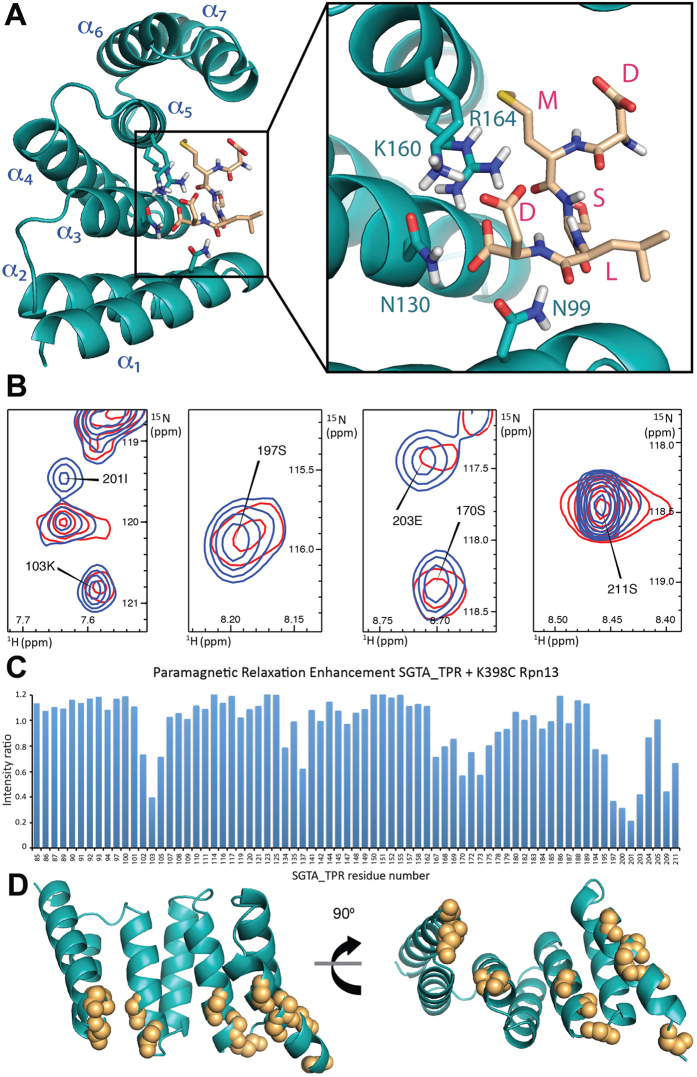
Modelling the SGTA_TPR/Rpn13 complex. (**A**) Model of SGTA_TPR/DMSLD peptide complex generated using the HADDOCK approach, with inset showing SGTA_TPR residues Asn-99, Asn-130, Lys-160 and Arg-164 that form a network of electrostatic interactions with the terminal aspartate on the DMSLD peptide. (**B**) Regions of the ^1^H-^15^N HSQC spectra of ^15^N-labelled SGTA_TPR upon binding to either the diamagnetic (dMTSL) probe labelled K398C Rpn13_260-407_ mutant (blue), or in the presence of the paramagnetic (MTSL) probe labelled K398C Rpn13_260-407_ mutant (red). (**C**) Paramagnetic relaxation enhancement (PRE) intensity ratios of SGTA_TPR residues upon binding to the spin labelled K398C Rpn13_260-407_ mutant. (**D**) Orthogonal cartoon views of SGTA_TPR (PDB accession code 2VYI), showing SGTA_TPR residues affected by intermolecular PRE induced line broadening upon binding to the K398C Rpn13_260-407_ mutant. Backbone amides within 20 Å of Rpn13_260-407_ residue 398 are shown as orange spheres.

Using CSP experiments combined with available mutagenesis data, an initial model of a 1:1 complex was generated by a HADDOCK based semi-rigid, data-driven approach using the DMSLD pentapeptide coordinates from the solution NMR structure of Rpn13 (PDB accession code: 2KR0) and the crystal structure of SGTA_TPR (PDB accession code: 2VYI). The three lowest energy HADDOCK clusters of the SGTA_TPR/DMSLD complex were analysed (Figure S7 and Table S1). The SGTA_TPR residues present at the binding interface with the DMSLD peptide are Asn-99, Asn-130, Lys-160 and Arg-164, with Lys-160 and Arg-164 clamping the two carboxylates present on the terminal aspartate of the DMSLD peptide (Fig. 4A). To determine the region of entry of the Rpn13′s C-terminal extension into the concave surface of the TPR domain in the SGTA_TPR/Rpn13_260-407_ complex, and hence the orientation of the DMSLD peptide in the TPR groove, a site-directed paramagnetic spin-label (MTSL) was incorporated via a K398C mutant of Rpn13. Proximity dependent line broadening of ^15^N-labelled SGTA_TPR backbone amide signals was monitored for intermolecular paramagnetic relaxation enhancement (PRE) effects upon addition of MTSL labelled K398C Rpn13_260-407_ (Fig. 4B). Significant line broadening effects were observed upon binding in backbone amide peaks of SGTA_TPR residues Met-102, Lys-103, Glu-105, Lys-137, Ser-170, Asn-173, Ser-197, Lys-200, Ile-201, Glu-203, Ser-211, all present on the lower edge of its concave surface lining the entry to the central groove (Fig. 4C,D). This finding suggests that the paramagnetic probe on K398C Rpn13_260-407_ is within a 20 Å distance of these residues, and when taken together with CSP data (Fig. 3C), confirms that the Rpn13_260-407_ C-terminal extension enters the SGTA_TPR groove via this lower edge of its concave surface, thus confirming the orientation of the DMSLD peptide within the groove.

### The SGTA-Rpn13 interaction influences steady state MLP levels

**Figure 5:**
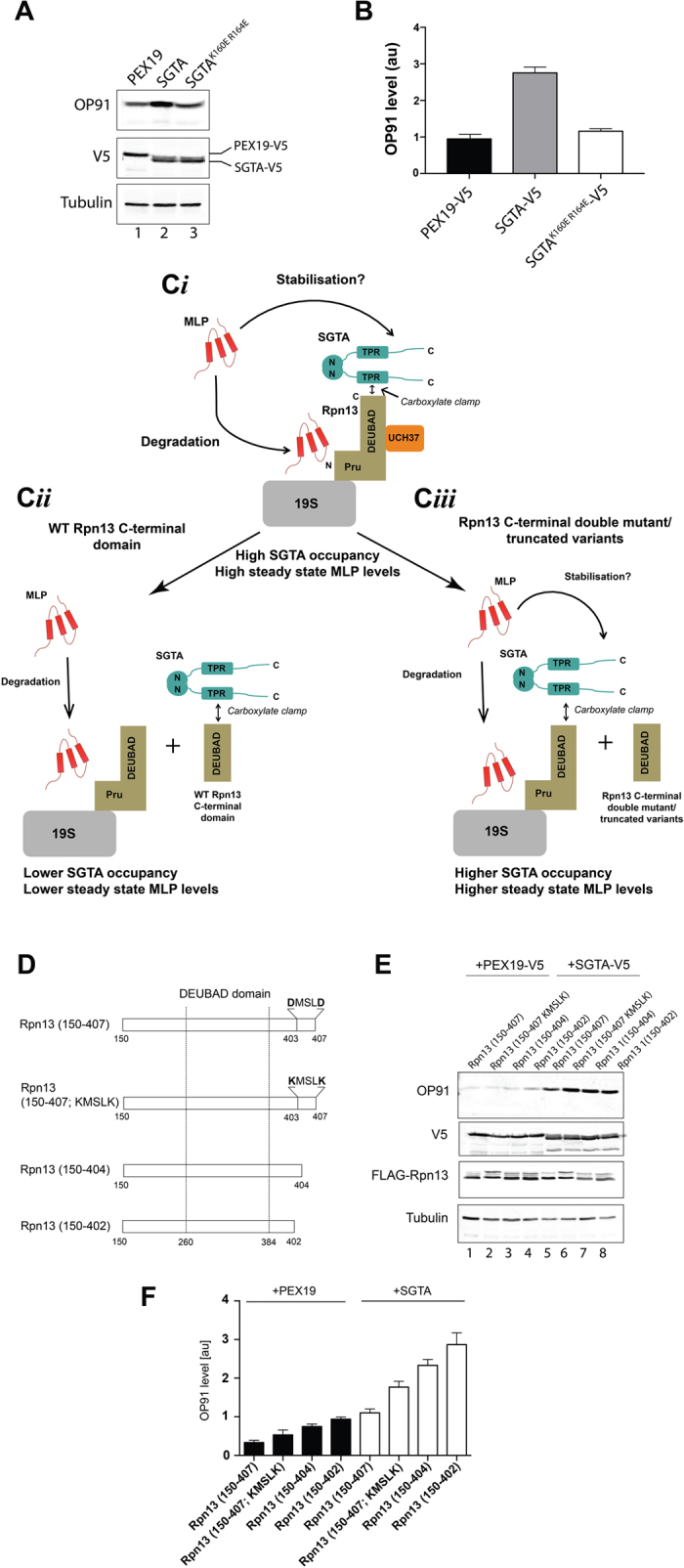
The SGTA-Rpn13 interaction alters steady state levels of a model MLP. (**A**) HeLa TRex Flp-In cells stably expressing OP91 after induction were transfected with plasmids for PEX19-V5, SGTA-V5 or the K160E/R164E SGTA-V5 mutant with a defective carboxylate clamp as indicated. After 24 h, OP91 expression was induced and the cells were grown for a further 24 h. Cells were then harvested directly into sample buffer and analysed by western blotting using primary antibodies to the N-terminus of opsin (OP91), the V5 tag (PEX19 and SGTA) or tubulin (loading control) in combination with secondary antibodies labelled with infrared dyes (LiCor) in order to produce quantitative signals as previously described^24^. (**B**) OP91 signals were quantified (see Materials and Methods) and normalised to the tubulin loading control, values show standard errors for n = 3. (**C**) Summary of the cell based assay employed to look at the *in vivo* effects of the Rpn13 variants. (**C*****i***) A schematic for the recruitment of exogenous SGTA to the Rpn13 subunit of the proteasome via a carboxylate clamp mechanism indicating its effect on steady state MLP levels. We speculate that SGTA recruitment may lead to a delay in the proteasomal degradation of MLPs, as denoted by “stabilisation”^5^. (**C*****ii***) The predicted scenario for the co-expression of a C-terminal fragment of Rpn13 that is lacking the PRU domain, and therefore unable to bind the proteasome, but can compete for binding to exogenous SGTA. (**C*****iii***) The predicted scenario for the co-expression of C-terminal fragments of Rpn13 that are defective in binding to the carboxylate clamp of SGTA. (**D**) Outline of Rpn13 C-terminal domain constructs used in co-expression assays. These include a KMSLK double point mutant and two C-terminally truncated variants, all targeting the carboxylate clamp mechanism. (**E**) HeLa TRex Flp-In cells (see **A**) were co-transfected with plasmids for PEX19-V5 (control; lanes 1–4) or SGTA-V5 (lanes 5–8) together with the Rpn13 variants indicated and further processed as described for (**A**) with the addition of the detection of Rpn13 fragments via an N-terminal FLAG tag. (**F**) OP91 signals were quantified as described for (**B**), values show standard errors for n = 3.

A previous study found that the overexpression of exogenous SGTA delays the proteasomal degradation of a model mislocalized membrane protein (MLP)^5^. Hence, SGTA overexpression typically increases the steady-state levels of model MLPs, including OP91, an N-terminal fragment of the polytopic integral membrane protein opsin that is inefficiently targeted to the endoplasmic reticulum^4^,^5^,^24^. However, the ability of exogenous SGTA to enhance steady state MLP levels is offset when key residues of the carboxylate clamp, located in its TPR domain, are mutated^24^. We recapitulated this experiment and found that the co-expression of exogenous SGTA-V5 led to a three-fold increase in OP91 levels when compared to the expression of either the K160E/R164E SGTA_TPR mutant or a PEX19 control (Fig. 5A,B; cf. Fig. 3E). These finding are consistent with the proposal that a TPR based interaction of exogenous SGTA contributes to its effects on steady state MLP levels^24^. In order to establish whether the carboxylate clamp mediated binding of exogenous SGTA to the Rpn13 subunit of endogenous proteasomes (see Fig. 5Ci) contributed to the enhanced levels of OP91 that we observe (Fig. 5A,B; see also Leznicki *et al*.^24^) we exploited a competition assay based on the co-expression of a C-terminal fragment of Rpn13 that lacks the N-terminal PRU domain responsible for binding to the proteasome^24^. In this scenario, the co-expression of an exogenous Rpn13 fragment that can bind to SGTA should compete for it, and thereby reduce the occupancy of SGTA on endogenous, proteasome associated Rpn13 (see Fig. 5Cii). The co-expression of such an Rpn13 fragment has previously been shown to reverse the effect of exogenous SGTA overexpression resulting in a lowering of steady state MLP levels^24^. In contrast, we hypothesized that the overexpression of an Rpn13 fragment that is defective in binding to SGTA will compete less effectively for it, and hence the occupancy of exogenous SGTA on endogenous proteasomal Rpn13 would be increased and steady state MLP levels would go up (Fig. 5Ciii). In order to explore the importance of the carboxylate clamp to the interaction of Rpn13 with SGTA we compared the effects of a previously described Rpn13 fragment incorporating the DEUBAD domain, and its C-terminal extension (residues 150–407; Rpn13_150-407_)^24^, to two truncated variants (residues 150–404; Rpn13_150-404_ and residues 150–402; Rpn13_150-402_) and a double point mutant (D403K/D407K; Rpn13_150-407_ KMSLK) all targeting the SGTA binding region of Rpn13 that we identify in this study (Fig. 5D). When the steady state OP91 levels were analyzed in the presence of exogenous SGTA, we found that all three Rpn13 fragments with altered C-termini were less effective than the unaltered Rpn13 C-terminal fragment at countering the SGTA mediated increase in the steady state level of OP91 (Fig. 5E,F). The most striking difference was a ~ three-fold increase in OP91 levels upon co-expression of Rpn13_150-402_ as compared to the Rpn13_150-407_ control (Fig. 5E, cf. lanes 5 and 8; Fig. 5F). Furthermore, although steady state OP91 levels are much lower in the absence of exogenous SGTA (Fig. 5E, cf. OP91 signal in lanes 1 to 4 and 5 to 8), quantification shows a similar trend when the different Rpn13 fragments are co-expressed with a PEX19 control. These data are consistent with the proposal that the binding of endogenous SGTA to the Rpn13 subunit of the proteasome may also influence the degradation of MLPs as previously suggested^24^. We conclude that the ability of exogenous SGTA to enhance the steady state levels of the model MLP, OP91, depends on its carboxylate clamp mediated binding to the C-terminal DMSLD pentapeptide region of Rpn13.

## Discussion

In this study, we characterize molecular-level details underlying the association of SGTA with the proteasome via the last five amino acids of the intrinsic proteasomal ubiquitin receptor Rpn13. We identify that this interaction occurs through a carboxylate clamp-mediated recognition event by the SGTA_TPR domain. We measure ITC binding affinity between SGTA_TPR and Rpn13 (both the isolated pentapeptide and much larger C-terminal domain, Rpn13_260-407_), which yield K_d_ values on the tens of micromolar scale, consistent with our NMR titration examined from the perspective of amides in Rpn13. Intriguingly the reciprocal titration defied saturation seemingly implying a weaker affinity than we have measured. We note that the K_d_ values we observe using ITC are consistent with other reported values for TPR domains interacting with carboxylate clamps from Hsp70 and Hsp90 chaperones^31^,^34^. Unfortunately, previous NMR-based studies of similar systems do not include the CSP spectra so we are unable to make a comparison with our own data. From our paramagnetic relaxation enhancement studies we observe that the Rpn13 peptide samples a lot of space in the TPR binding pocket and speculate that this could have some effect on the way that the binding is observed from the TPR perspective in the titration.

In a cellular context, our data support the suggestion that this mode of binding is responsible for the recruitment of SGTA to the proteasome (cf. Fig. 5Ci). In the case of endogenous SGTA, the precise significance of this interaction remains to be established. However, when exogenous SGTA is overexpressed this results in an increase in the steady state level of model MLPs^24^ potentially through a delay in MLP degradation at the proteasome^5^. Our current study suggests that the effect of exogenous SGTA overexpression on steady state MLP levels requires its binding to the Rpn13 subunit of the proteasome. Hence, the overexpression of an SGTA mutant with a defective carboxylate clamp largely negates the effect of SGTA overexpression on steady state OP91 levels (cf. Fig. 5B,F). The same effect is observed upon co-expression of an Rpn13 fragment that competes for binding to exogenous SGTA, but is missing the N-terminal PRU domain of Rpn13 so it can no-longer bind to the proteasome.

Since SGTA exists as a homodimer, its individual TPR domains could, for example, interact with both Rpn13 and Hsp70/90 molecular chaperones, respectively^19^,^24^,^33^. It is evident that Hsp70/90 interacts with SGTA via a similar two-carboxylate clamp mechanism forming a comparable network of electrostatic interactions with residues in the TPR groove involving main chain and side chain carboxylates on their terminal aspartate residue^18^. Therefore, SGTA-bound substrates might gain proximity to these ATP-dependent molecular chaperones whilst also associated with the proteasome. Furthermore, although MLPs delivered to the proteasome are likely ubiquitinated through the actions of the BAG6 complex^11^,^13^, SGTA appears to promote their deubiquitination^4^,^5^. In this context, the Rpn13 dependent activation of the UCH37 deubiquitinase is noteworthy (cf. Fig. 5Ci)^26^,^27^, since it would provide SGTA-bound substrates with an opportunity for selective deubiquitination^24^. Since BAG6 interacts with the Rpn10 proteasomal receptor^12^,^23^, which is in close proximity to the Rpn13 subunit on the 19S regulatory particle, it has been suggested that a BAG6/SGTA dependent cycle of substrate ubiquitination and deubiquitination may occur at the proteasome^24^, in addition to its operation in the cytosol^4^,^5^. Whatever the physiological role of the interaction between Rpn13 and SGTA, we show that their binding is mediated through a well-defined carboxylate clamp mechanism and identify key residues in both components that enable them to selectively associate.

## Materials and Methods

### Plasmid preparation

Gene fragments encoding human Rpn13 (260–407 and 260–402) and SGTA_TPR domain (84–211) were PCR amplified from cDNA (Life Technologies) and cloned into the BamHI/XhoI site of a modified pET28 vector which encodes an N-terminal thioredoxine A (TxA) fusion protein followed by a hexahistidine tag and tobacco etch virus (TEV) protease cleavage site. The SGTA_TPR double mutant (K160E/R164E) and Rpn13 C-terminal cysteine mutant (K398C) were obtained by PCR mutagenesis reactions using pET28-TxA-SGTA_TPR (84–211) and pET28-TxA-Rpn13 (260–407) vectors, respectively, as templates and different oligonucleotides carrying the mutated codons. The plasmids for the overexpression of SGTA-V5, SGTA^K160E/R164E^-V5 PEX19-V5 and the FLAG tagged mouse Rpn13 (150–407) variant in NpFLAG-CMV2 were previously described^24^. Site directed mutagenesis was used to remove the C-terminal 3 and 5 residues from this Rpn13 fragment, generating Rpn13 (150–404) and Rpn13 (150–402) respectively, and to create the Rpn13 (150–407; KMSKL) mutant. All constructs were validated by DNA sequencing prior to use.

### Protein expression and purification

SGTA_TPR and Rpn13 plasmids were transformed into *E. coli* BL21(DE3) Rosetta cells, with protein expression induced by adding 0.3–0.5 mM isopropyl-β-D-thiogalactopyranoside (IPTG) to bacterial cultures in LB media at OD_600_ ≈ 0.8, followed by overnight incubation at 18 °C. For isotopically labelled proteins, growth was carried out in M9 minimal media supplemented with labelled ammonium chloride (>98% ^15^N, Sigma-Aldrich). Harvested cells were resuspended in lysis buffer (20 mM potassium phosphate, pH 8.0, 300 mM NaCl, 10 mM Imidazole, 250 μM TCEP), supplemented with protease inhibitor tablets (complete mini, EDTA-free, Roche) and 0.5 mM PMSF and lysed by sonication or by cell disruption (Constant Systems Ltd). Cell membranes and insoluble material were removed by centrifugation and soluble fractions were purified using metal affinity chromatography (HisTrap^TM^ HP 5 ml, GE Healthcare). Recombinant proteins were eluted with buffer containing 300 mM imidazole, then dialyzed against cleavage buffer (20 mM potassium phosphate, pH 8.0 and 300 mM NaCl) and simultaneously digested with homemade TEV protease (≈100 μg/ml) at 4 °C overnight. After cleavage a second metal affinity chromatography step was carried out to remove tags, undigested protein and TEV protease; with the desired protein recovered in the flow through then loaded on to a HiLoad 16/60 Superdex 75 column (GE Healthcare) at a flow rate of 1 ml/min, pre-equilibrated in 10 mM potassium phosphate pH 6.0, 100 mM NaCl and 250 μM TCEP buffer, for a final size-exclusion chromatography (SEC) step. Proteins were concentrated using Vivaspin concentrators (Sartorius Stedin) and sample purity and homogeneity was assessed by SDS-PAGE, mass spectrometry and NMR. The Rpn13 derived DMSLD peptide used in this study was purchased from Alta BioScience (Birmingham, UK) with the purified peptide verified by HPLC and mass spectrometry.

### NMR Spectroscopy

Protein samples at concentrations between 300 μM and 1 mM were prepared in 10% D_2_O (Sigma Aldrich), 10 mM potassium phosphate pH 6.0, 100 mM NaCl and 250 μM TCEP buffer (with 10 μM DSS for proton chemical shift referencing). All NMR experiments were acquired in 5 mm NMR tubes at 25 °C on 500 MHz and 700 MHz Bruker Avance spectrometers equipped with cryoprobes and operated by the TopSpin 3.1 software package. All NMR spectra were processed with TopSpin and analysed with CcpNMR Analysis^35^. Proteins used for NMR titrations were dialysed overnight against the same buffer (10 mM potassium phosphate pH 6.0, 100 mM NaCl and 250 μM TCEP) and mixed in different molar ratios, with the concentration of the labelled protein maintained constant. ^1^H-^15^N HSQC experiments were recorded for each titration point at 25 °C. Chemical shift perturbation (CSP) values were calculated for each amide signal using the following formula:


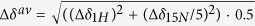


where Δδ_1H_ and Δδ_15N_ are the chemical shift differences for the same amide in its free and bound state (δ_free_−δ_bound_) and for proton and nitrogen chemical shift values respectively. CSP results were mapped on to the SGTA_TPR crystal structure (PDB code 2VYI) using PyMOL. ^1^H-^15^N heteronuclear NOE experiments were performed with a four second saturation transfer or control period at 700 MHz. Sample concentrations of ^15^N-labelled Rpn13_260-407_ were maintained at 350 μM both in its free and bound state with unlabelled SGTA_TPR at a 1:1 molar ratio.

### Paramagnetic relaxation enhancement experiments

The Rpn13 C-terminal residue Lys 398 was mutated to a cysteine for paramagnetic spin labelling studies. Mutant Rpn13_260-407_ was treated with excess TCEP for 2 hours to ensure cysteines were reduced, followed by dialysis against 10 mM potassium phosphate pH 6.0, 100 mM NaCl buffer to remove excess TCEP. Mutant samples were then incubated with either diamagnetic 1-acetyl-2,2,5,5-tetramethyl-Δ3-pyrroline-3-methyl methanethiosulfonate (dMTSL) or paramagnetic 1-oxyl-2,2,5,5-tetramethylpyrroline-3- methyl methanethiosulfonate (MTSL) spin labels (Santa Cruz Biotechnology) overnight at 4 °C. Excess dMTSL/MTSL was removed by extensive dialysis against 10 mM potassium phosphate pH 6.0, 100 mM NaCl. ^15^N-labelled SGTA_TPR in 10 mM potassium phosphate pH 6.0, 100 mM NaCl buffer was mixed with each spin labelled Rpn13 sample at a 1:1.2 ratio. A ^1^H-^15^N HSQC spectrum was acquired for each 200 μM sample with a 1 hour acquisition.

### ITC

ITC experiments were performed using an ITC-200 microcalorimeter from Microcal (GE Healthcare) at 25 °C following the standard protocol as reported previously^7^. Proteins or peptide samples were prepared in 10 mM potassium phosphate pH 6.0, 100 mM NaCl, 250 μM TCEP. In each titration, either 40 injections of 1 μL or 20 injections of 2 μL of SGTA_TPR each, at a concentration of 1 mM, were added to a sample of Rpn13 or the Rpn13 derived DMSLD peptide at a concentration of 50 μM in the reaction cell. A nonlinear least-squares minimization algorithm was applied to integrated heat data obtained for the titrations, corrected for heats of dilution, in order to fit the experimentally obtained values to a theoretical titration curve. This was performed using the MicroCal-Origin 7.0 software package. ΔH (reaction enthalpy change in kcal/mol), K_b_ (equilibrium binding constant in per molar), and n (molar ratio between the proteins in the complex) were used as fitting parameters. The reaction entropy, ΔS, was calculated using the relationships ΔG = −RT lnK_b_ (*R* = 8.314 J/(mol K), T 298 K) where ΔG = ΔH − TΔS. Dissociation constants (*K*_d_) have been determined for each interaction. Binding was assumed to be at one site (n = 1), to determine the binding affinity (K_d_) and thermodynamic parameters.

### SGTA_TPR/DMSLD peptide complex assembly using HADDOCK

CSP studies combined with mutagenesis experiments defined clear interaction surface areas in the SGTA_TPR/DMSLD peptide complex. These data were used to generate a model of this protein-peptide complex using the HADDOCK approach^36^,^37^. For the calculation, the PDB-deposited structure of SGTA_TPR (2VYI) and the coordinates of the DMSLD pentapeptide from the solution NMR structure of Rpn13 (2KR0) were used. Ambiguous Interaction Restraints (AIRs) were implemented according to the standard HADDOCK protocol. CSP experiments allowed identification of 10 amino acid residues in SGTA_TPR and 3 within the DMSLD peptide with chemical shift changes greater than 0.10 ppm. The Naccess program was used to determine relative solvent accessibility, and residues with higher that 45% values were identified as active. Based on this approach, 10 residues in SGTA_TPR and 3 in the DMSLD peptide, were identified as active. These were SGTA_TPR residues 99, 102, 107, 130, 133, 161, 163, 168, 171, 198 and Rpn13 residues 405, 406 and 407 (for the DMSLD peptide). Solvent exposed residues juxtaposed to active residues were automatically designated as passive residues. Rigid body energy minimization was used to generate one thousand initial complex structures, from which the best 200 (lowest total energy) were selected for torsion angle and Cartesian dynamics in an explicit water solvent. Default scaling was applied for energy terms. Following the standard protocol, cluster analysis generated 130 structures in 11 cluster ensembles. The top-scoring cluster (lowest energy) was considered as the most reliable result as determined by HADDOCK benchmark testing.

### Effects of co-expressing SGTA and Rpn13 fragments on steady state MLP levels

The effect of overexpressing plasmids encoding a PEX19-V5 control, SGTA-V5 or a K160E/R164E SGTA-V5 mutant with a defective carboxylate clamp were determined using a TRex Flp-In HeLa cell line stably expressing the model MLP OP91 under the control of a tetracycline-inducible promoter^24^. Cells were transfected with the relevant plasmids as indicated (Fig. 5A,B) and then grown for 24 h, OP91 expression was then induced with 1 μg/ml tetracycline and growth continued for a further 24 h. Cells were then harvested directly into sample buffer and analysed by western blotting using an opsin primary antibody in combination with secondary antibodies labelled with infrared dyes (LiCor) in order to produce quantitative signals as previously described^24^. For analysis of Rpn13-fragments, cells were co-transfected with plasmids encoding PEX19-V5 or SGTA-V5 and one of four FLAG-Rpn13 (150–407) variants as indicated (Fig. 5D) and grown for 24 h before OP91 expression was induced with 1 μg/ml tetracycline and growth continued for a further 24 h. Cells were then harvested directly into sample buffer and analysed by quantitative western blotting as described above^24^. Quantifications show the average of three biological repeats with standard error (Fig. 5B,F). The OP91 signals were quantified using Odyssey 2.1 software, normalised to the tubulin loading control, and plotted using GraphPad Prism 7.0 software. All antibodies are as previously described^24^.

## Additional Information

**How to cite this article**: Thapaliya, A. *et al*. SGTA interacts with the proteasomal ubiquitin receptor Rpn13 via a carboxylate clamp mechanism. *Sci. Rep*. **6**, 36622; doi: 10.1038/srep36622 (2016).

**Publisher’s note:** Springer Nature remains neutral with regard to jurisdictional claims in published maps and institutional affiliations.

## Supplementary Material

Supplementary Information
