# Supplementary material for: SGTA interacts with the proteasomal ubiquitin receptor Rpn13 via a carboxylate clamp mechanism
Source: Sci Rep. 2016 Nov 9;6:36622. doi: 10.1038/srep36622 (PMC5101480; doi:10.1038/srep36622)
Supplement: Supplementary Information [file srep36622-s1.pdf]

Supplementary Information

SGTA interacts with the proteasome via a carboxylate clamp mechanism

Arjun Thapaliya[1], Yvonne Nyathi[2], Santiago Martínez-Lumbreras[1], Ewelina M. Krysztofinska[1], Nicola J. Evans[1], Isabelle L. Terry[1], Stephen High[2] & Rivka L. Isaacson[1†]

[1]Department of Chemistry, King's College London, Britannia House, 7 Trinity Street, London, SE1 1DB, U.K.

[2]Faculty of Life Sciences, University of Manchester, The Michael Smith Building, Oxford Road, Manchester, M13 9PT, U.K.

[†]Corresponding author; e-mail: rivka.isaacson@kcl.ac.uk telephone: +4420 7848 7338

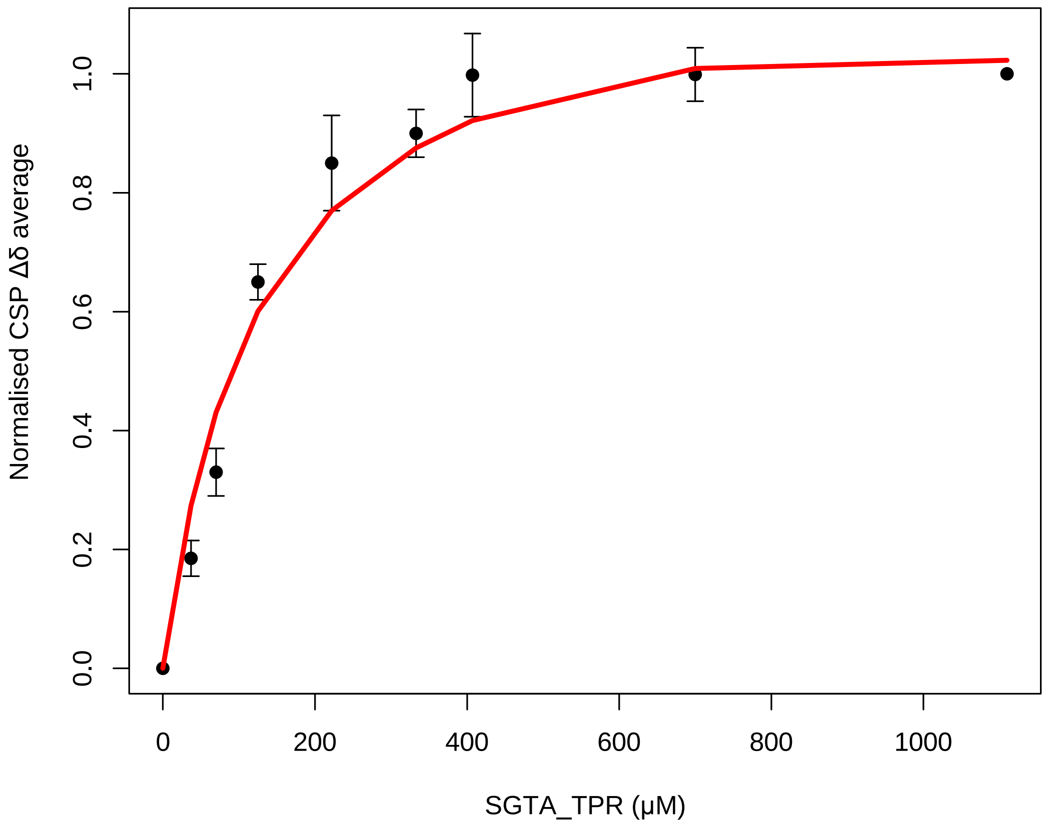

Figure S1. Normalised CSP data of the most perturbed Rpn13$_{260\text{-}407}$ residues (405, 406, 407) upon titration with different concentrations of SGTA_TPR. NMR titrations were carried out at varying molar ratios (1:0, 1:0.1, 1:0.2, 1:0.3, 1:0.6, 1:0.9, 1:1.1, 1:1.9 and 1:3). Fitting was carried out using DynaFit to give a K_d of 3.27 ± 0.87 μM.

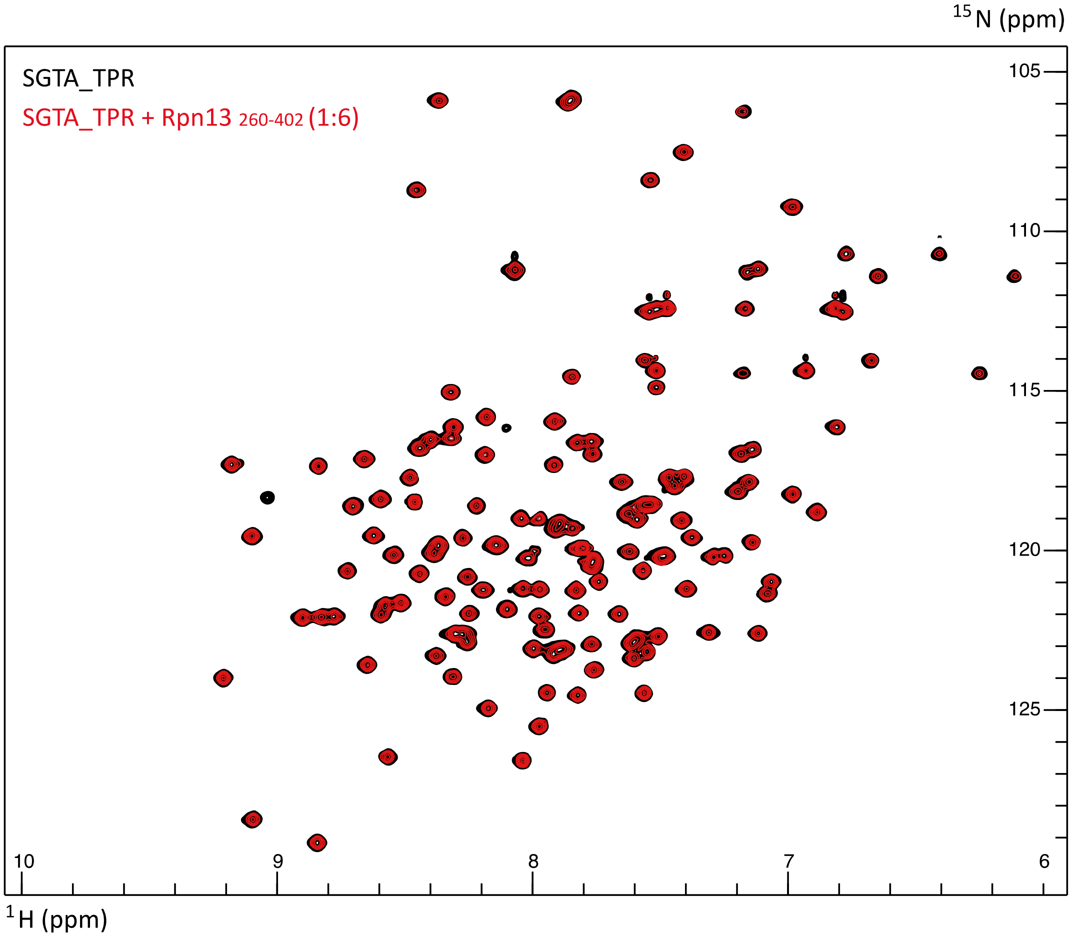

Figure S2. Rpn13 devoid of its extreme C-terminal residues 403-407 does not interact with SGTA_TPR. ^{1}H-^{15}N HSQC spectra of ^{15}N-labelled SGTA_TPR in its free form (black), and with a six-fold molar excess of unlabelled $Rpn13_{260-402}$ (red).

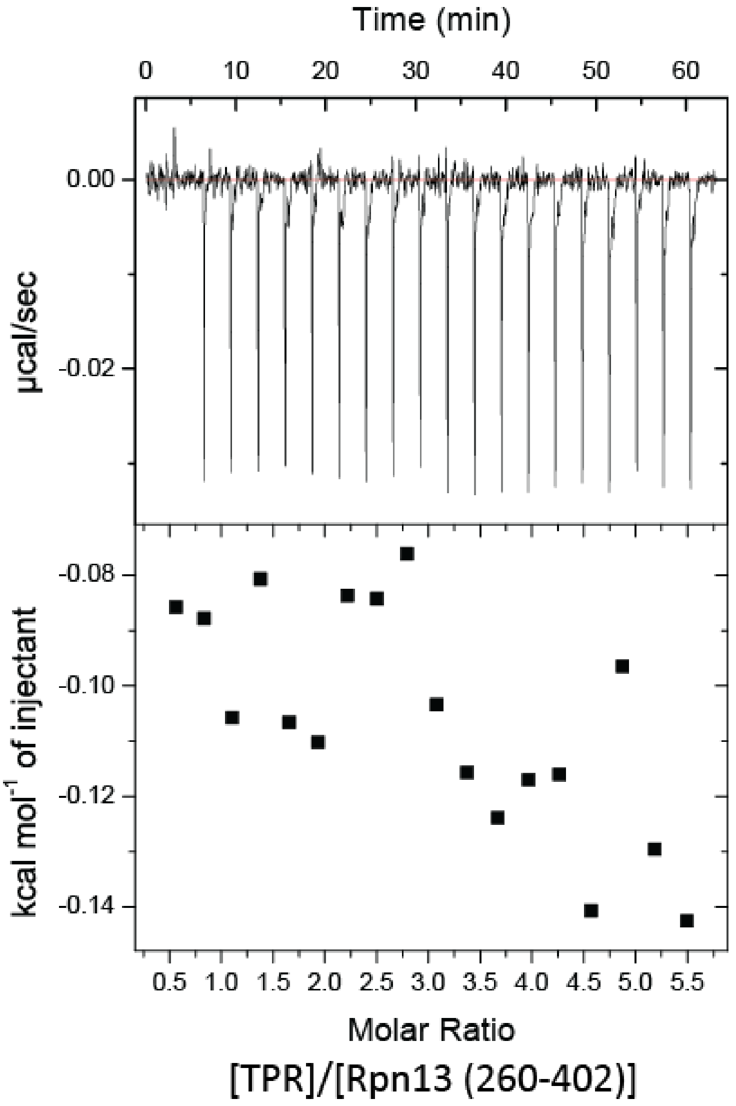

Figure S3. ITC binding assay showing that Rpn13$_{260\text{-}402}$ does not interact with SGTA_TPR. Binding isotherm showing 20 injections of 2 μL of SGTA_TPR at a concentration of 1 mM into a calorimeter cell that contained 50 μM Rpn13$_{260\text{-}402}$.

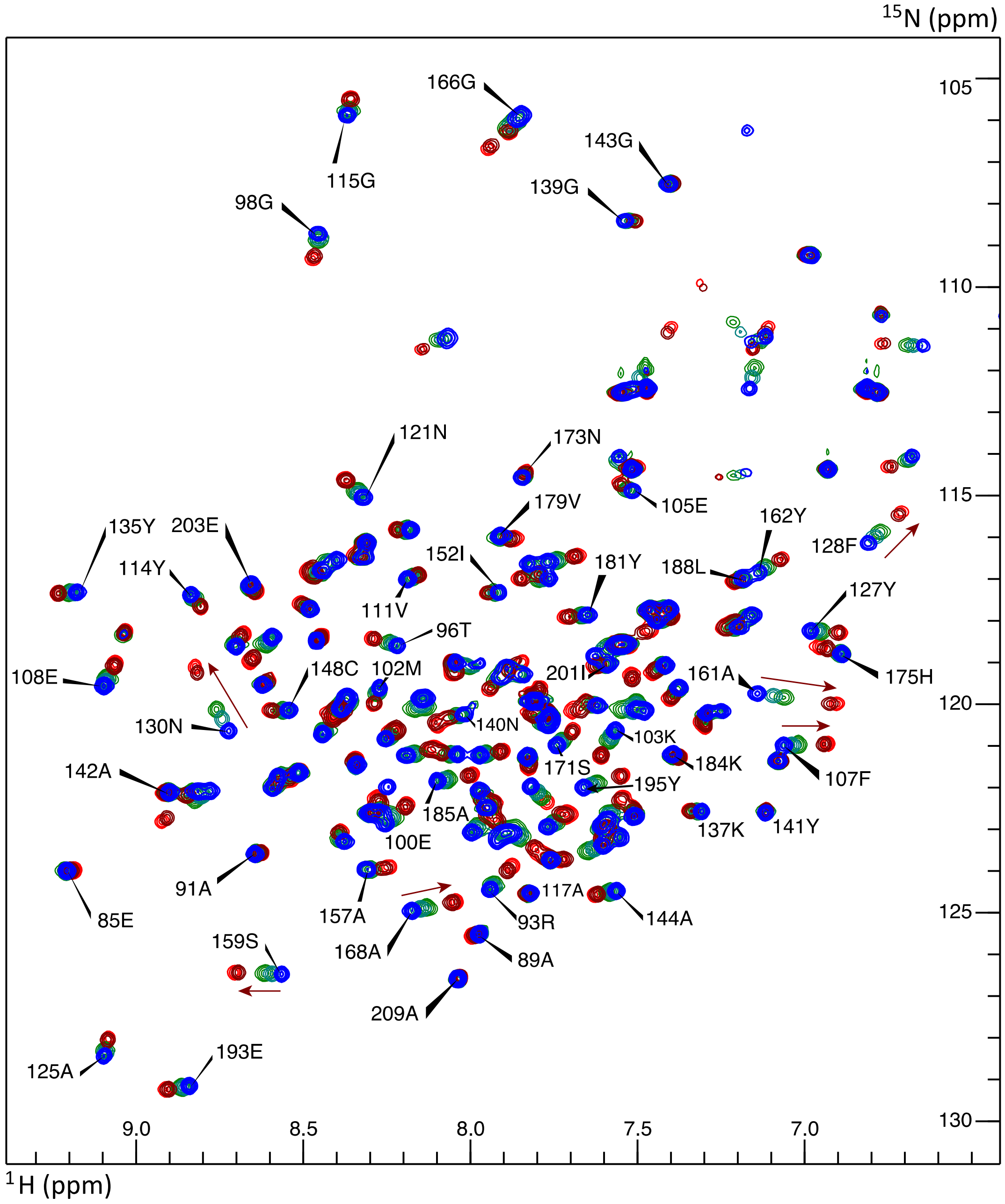

Figure S4. The Rpn13 derived DMSLD peptide is necessary and sufficient for its interaction with SGTA_TPR. ^{1}H-^{15}N HSQC spectra of ^{15}N-labelled SGTA_TPR at different titration points with unlabelled Rpn13 extreme C-terminal derived DMSLD pentapeptide (1:0, 1:0.5, 1:1, 1:6, 1:8; in blue, teal, green, maroon, and red respectively).

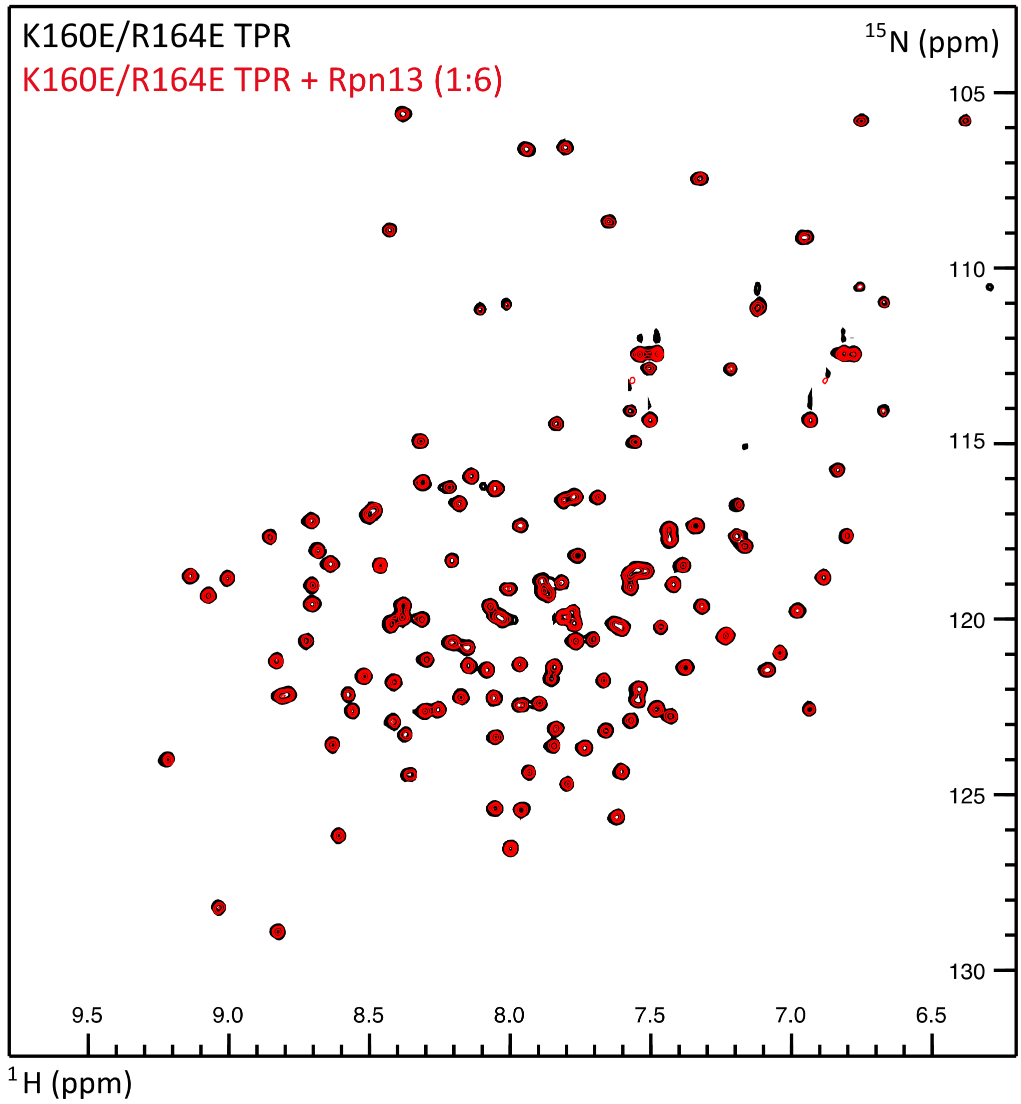

Figure S5. K160E/R164E double mutant SGTA_TPR and Rpn13$_{260\text{-}407}$ do not interact. ^{1}H-^{15}N HSQC spectra of ^{15}N-labelled K160E/R164E SGTA_TPR in its free form (black), and with a six-fold molar excess of unlabelled Rpn13$_{260\text{-}407}$ (red).

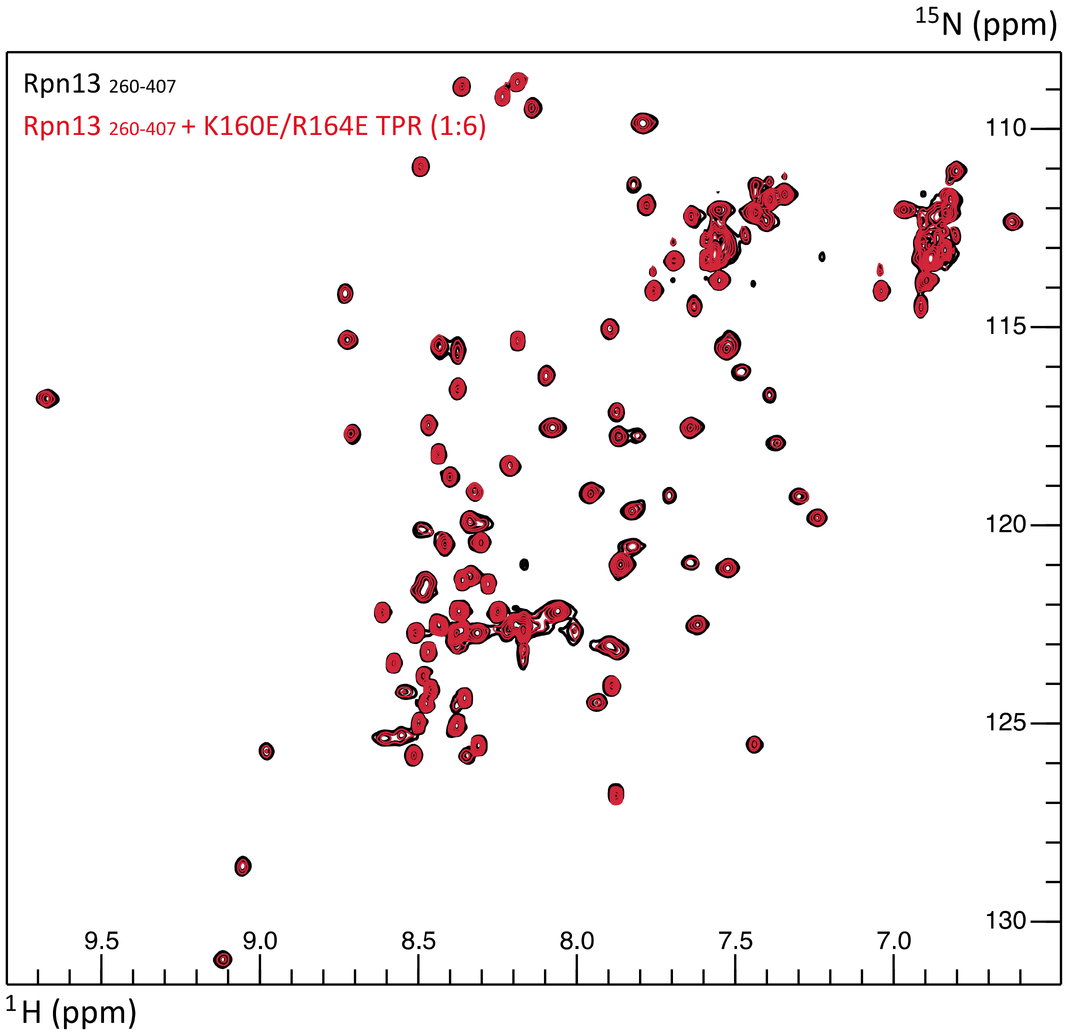

Figure S6. Rpn13$_{260-407}$ and K160E/R164E double mutant SGTA_TPR do not interact. ^{1}H-^{15}N HSQC spectra of ^{15}N-labelled Rpn13$_{260-407}$ in its free form (black), and with a six-fold molar excess of unlabelled K160E/R164E SGTA_TPR (red).

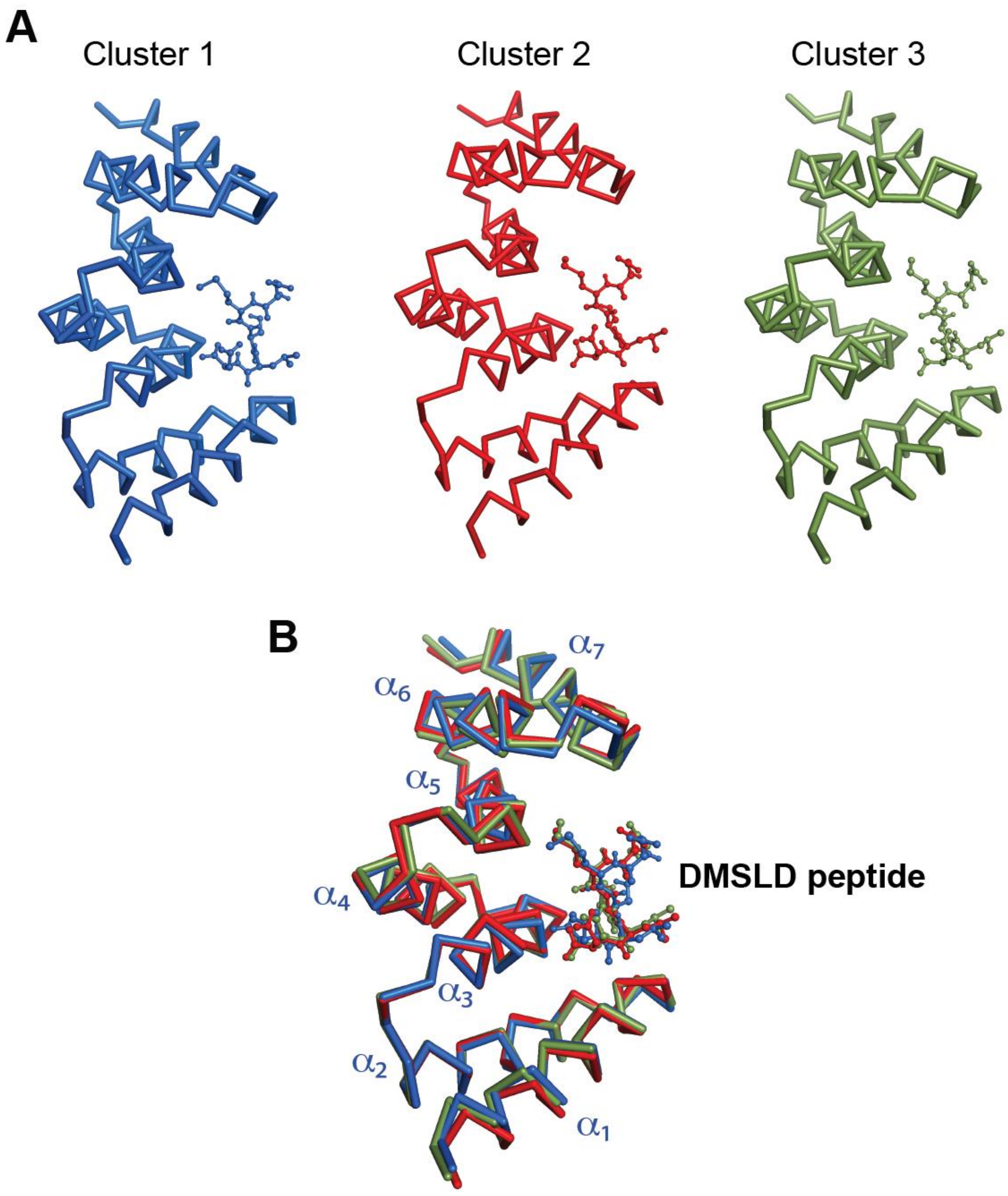

Figure S7. SGTA_TPR/DMSLD peptide complex HADDOCK modelling results. A) Three top scoring clusters of the SGTA_TPR/DMSLD complex generated by HADDOCK. B) Overlay of HADDOCK-generated top scoring clusters of the SGTA_TPR/DMSLD complex showing the DMSLD peptide bound to the SGTA_TPR groove.

Supplementary tables

Cluster	HADDOCK score	Cluster size	RMSD (Å)	Van der Waals energy (kcal/mol)	Restraints violation energy (kcal/mol)	Buried surface area (Å^2)	Electrostatic energy (kcal/mol)	Desolvation energy (kcal/mol)	Z score
Cluster1	-107.6 +/-2.1	40	3.6 +/- 0.1	-21.1 +/- 4.8	33.4 +/- 17.89	1119.1 +/- 63.2	-545.0 +/- 24.0	19.2 +/- 4.9	-1.8
Cluster2	-98.8 +/- 6.5	5	1.0 +/- 0.1	-17.8 +/- 5.8	97.8 +/- 19.63	1234.2 +/- 12.8	-533.0 +/- 37.4	15.7 +/- 1.5	-1.4
Cluster3	-92.2 +/- 18.5	4	3.9 +/- 0.1	-22.7 +/- 2.8	38.9 +/- 20.28	1009.9 +/- 73.8	-455.2 +/- 93.0	17.7 +/- 2.7	-1.1

Table S1: Energetic parameters obtained for the three best clusters of SGTA_TPR/DMSLD peptide complex calculated using HADDOCK.
